# Examining young adults daily perspectives on usage of anxiety apps: A user study

**DOI:** 10.1371/journal.pdig.0000185

**Published:** 2023-01-26

**Authors:** Andreas Balaskas, Stephen M. Schueller, Anna L. Cox, Chuck Rashleigh, Gavin Doherty

**Affiliations:** 1 School of Computer Science and Statistics, Trinity College Dublin, Dublin, Ireland; 2 Department of Psychological Science, University of California, Irvine, Irvine, California, United States of America; 3 UCLIC, University College London, London, United Kingdom; 4 Student Counselling Services, Trinity College Dublin, Dublin, Ireland; ISI Foundation: Fondazione ISI - Istituto per l’lnterscambio Scientifico, ITALY

## Abstract

The growing number of mental health smartphone applications has led to increased interest in how these tools might support users in different models of care. However, research on the use of these interventions in real-world settings has been scarce. It is important to understand how apps are used in a deployment setting, especially among populations where such tools might add value to current models of care. The objective of this study is to explore the daily use of commercially-available mobile apps for anxiety that integrate CBT, with a focus on understanding reasons for and barriers for app use and engagement. This study recruited 17 young adults (age M = 24.17 years) while on a waiting list to receive therapy in a Student Counselling Service. Participants were asked to select up to two of a list of three selected apps (Wysa, Woebot, and Sanvello) and instructed to use the apps for two weeks. Apps were selected because they used techniques from cognitive behavioral therapy, and offer diverse functionality for anxiety management. Qualitative and quantitative data were gathered through daily questionnaires to capture participants’ experiences with the mobile apps. In addition, eleven semi-structured interviews were conducted at the end of the study. We used descriptive statistics to analyze participants’ interaction with different app features and used a general inductive approach to analyze the collected qualitative data. The results highlight that users form opinions about the apps during the first days of app use. A number of barriers to sustained use are identified including cost-related issues, inadequate content to support long-term use, and a lack of customization options for different app functions. The app features used differ among participants with self-monitoring and treatment elements being the most used features.

## Author summary

In recent years, there has been an increased need for mental health services among students on college campuses. Several mobile applications have been developed to support mental health and expand treatment delivery methods to increase the reach of mental health care. Anxiety disorders are among the most common mental health problems affecting young adults and cognitive behavioral therapy (CBT) is considered the most common psychotherapeutic treatment of anxiety disorders. Even though there are a plethora of mental health apps for anxiety available, evidence suggests that apps are not used for a long period of time. Here, we conducted a two-week study with young adults who have contacted a mental health service and are waiting for treatment to explore their daily perspectives on the usage of commercially-available mobile apps for anxiety that integrate CBT with a focus on understanding the reasons for, and barriers to, engagement during app use. We found that users of such apps form opinions during the first days of use, opinions that remain consistent. We also found that many app features are not used often and the same apps are used differently by different people. Overall, our study provides new insights into the barriers and factors affecting long-term use at different time points during app use.

## 1. Introduction

Anxiety disorders are among the most common mental health problems affecting young adults (age 18–30 years). Several evidence-based treatments for anxiety exist, and clinical evidence suggests that relaxation training, CBT, and mindfulness can reduce anxiety symptoms [1–4]. While several evidence-based treatments for anxiety exist, cognitive behavioral therapy (CBT) is considered the most common psychotherapeutic treatment of anxiety disorders [5]. There has been an increasing number of young adults seeking support since the pandemic began [6]. Rates of mental health distress, including anxiety and loneliness, increased during the pandemic as closures and physical distancing disrupted school, work, and social activities [7,8]. However, access to traditional face-to- face therapy in many countries is limited by cost and availability [9–11]. The expanding need for mental health services among students on college campuses in the past few years has led to long waitlists for university counseling centers for students seeking treatment [12]. Counseling center staff and administrators are working together to find cost-effective options for supporting students [13–15].

Recent years have seen an increased development of internet and mobile-based solutions in research settings for expanding treatment delivery methods to increase the reach of mental health care [16,17]. Mental health applications have been developed across both academia and industry to deliver psychological interventions in daily life [18,19]. Mental health apps are software programs that can be accessed via the smartphone or mobile device and are used to deliver a range of guided or self-help intervention strategies, provide information about mental health, and enable real-time communication with health care professionals [18,19]. As a result of the high demand for mental health support and inadequate supply, there has been a rapid rise in investment in the mental health and wellness space [20]. This has resulted in an increase in the number of such apps available on the market. It is estimated that more than 10.000 commercially mental health apps are available [21]. Apps can support individuals in managing their own mental health, and can be used privately and “on the go” [22]. Even though there is a breadth of apps available, evidence suggests that many consumer mobile apps are inconsistent with evidence-based treatment [19,23–25], and often lack the involvement of health care professionals in their development [26]. Despite evidence supporting the effectiveness of certain mental health apps, many apps do not meet evidence standards [27]. Clinicians and adults awaiting support for their mental health recognize the need for alternative forms of intervention such as the use of apps which hold the potential to address barriers in mental health service delivery [15,28].

Despite the increased interest in using mental health apps in care settings, previous research has shown that there are high attrition rates among them [29,30]. Even though the number of app installs may seem high, apps are not often used daily nor for a long period of time [31,32], with evidence suggesting that most mental health apps are rarely used after being downloaded and only opened a few times [33]. Recent studies examining engagement with popular mental health apps from commercial marketplaces found that only 4% of users who downloaded a mental health app opened it again after 15 days [31], only a small number of apps have attracted a substantial number of active users, and 56% of anxiety apps had no monthly active users [32]. These variations in app usage indicate that we need to understand more about the interactions of users with different app features. In order to facilitate sustained app usage, we need to get more insight into user experiences [32].

Engagement is an important factor in supporting the sustained use of such apps. The concept of engagement is broad in nature as reflected in prior research reviewing and developing the concept of engagement across different contexts [34–36]. Engagement as a subjective experience in human–computer interface research has for example been characterized as feeling focused, attentive, and satisfied when using a digital technology [37]. In comparison, engagement in clinical research has been defined and associated with objective/behavioral metrics of use of or interactions with a mental health intervention, such as number of log-ins or time spent using the technology [31,38,39]. ‘Low engagement’, in the context of health services refers to a lack of uptake and/or poor adherence to an intervention among service users [33]. However, engagement also entails users’ subjective experience which may be a mechanism for change in clinical outcomes [38, 40].

Designing for longitudinal patient engagement is challenging [41,42]. Challenges of engagement may be associated with factors related to mental health conditions such as high variability in mood, often transient feelings of crisis, reduced motivation, the heterogeneity of symptoms experienced at the individual level, and low levels of app usability (apps being difficult or unenjoyable to use) [33]. Previous research has shown that greater engagement with digital interventions is significantly associated with improvements in mental health [43], and technology-supported strategies ranging from reminders, coaching, personalized information, and peer support appear to increase engagement [44]. However, there are no studies that systematically examine which app features or engagement metrics increase the sustained use of apps [43, 45] and the mechanism(s) through which such strategies promote engagement [44].

Previous research in clinical settings has focused primarily on examining the effective- ness and efficacy of an intervention as a whole [46,47]. In addition, Human-Computer Interaction (HCI) research has focused primarily on examining factors that affect user experience by involving participants during the design process or by collecting retrospec- tive data after app use. [48–53]. However, users’ perspectives on the daily real-world usage of such apps to investigate the reasons affecting attrition rates have not been explored yet.

User studies are valuable for understanding the underlying reasons why people may engage with some apps more than others [54]. The study described in this paper sheds light on specific engagement problems occurring during app use at particular time points from a users’ perspective and offers a resource for researchers and designers who want to deploy mental health apps in the future. It is important to understand how the target audiences for these products, for example those seeking treatment for mental illnesses, actually use these apps in their daily life. By doing this, we will be able to improve future apps and sustain their use. We explored one specific client group, those on waitlists for mental health treatments, as this has been a proposed use case for these tools to expand access to care [55,56]. The objectives of this study are thus to explore users’ perspectives on the daily use of commercially-available mobile apps for anxiety that integrate CBT, with a focus on understanding reasons for, and barriers to, engagement during app use.

## 2. Method

We conducted a user study in which people on a counseling center waitlist were asked to use mental health apps from a suggested list for two weeks. The apps were used to elicit further feedback and design requirements for apps targeting anxiety disorders and would not be assessed for efficacy. The set of candidate apps for inclusion was taken from a recent review of anxiety apps which examined how cognitive behavioral elements are delivered by anxiety apps, and their functionalities to support user engagement and tailoring based on user needs [57]. Detailed methods of app selection and assessment are outlined in [57]. Starting from this list of apps, we searched and selected science-backed apps that are actively used by users. We considered apps as being science-backed based on the existence of at least one published article that presents some evidence for effectiveness for anxiety management, with effectiveness interpreted as an app’s capability of reducing anxiety symptoms. Additionally, we considered apps as engaging based on available analytics data in relation to engagement metrics such as the number of yearly downloads, the number of daily active users (DAU), the number of monthly active users (MAU), and the number of active sessions worldwide and in the US market. We used a software tool (Apptopia) to gather analytic data showing the popularity of apps among stores based on these engagement metrics. This resulted in a total of three apps for inclusion in our study. The selected apps offer diverse functionality and features. Because app descriptions from research studies may not be exactly the same as those of the currently available product, we checked the apps available on the market for any inconsistencies. Detailed information on each app is presented in the S1 Appendix.

**Sanvello:** Sanvello (former Pacifica) is an app targeting the management of stress, anxiety, and depression [58]. Upon downloading the app, users select three goals they want to work on from a list of 8 options. Users receive recommended activities based on self-monitoring data. The app offers psychoeducation, meditation, relaxation exercises, cognitive reframing, and goal setting [58]. Social features provide professionals with access to a user’s profile, accompanied by a community feature comprising discussions and chat groups. Additionally, the app offers visualization of a variety of user inputs that allows the identification of factors contributing to specific events, and comparison of entries [58]. Gamification is implemented with the use of level upgrades based on interaction with different app features. A randomized controlled trial published in 2019 showed greater decreases in depression, anxiety, and stress, and increases in self-efficacy for the participants in the active condition [58].

**Wysa:** Wysa is an automated conversational agent aimed at building mental resilience and promoting mental well-being using a text-based conversational interface [59]. Users can select the areas they want to work on on the home screen. Additionally, the chatbot informed by self-monitoring data provides via written conversation evidence-based strategies such as cognitive re-framing, breathing exercises, and mindfulness [59]. An ‘SOS’ feature creates a safety plan and provides access to crisis helplines. By paying an additional fee, users can also connect with a licensed therapist through the app. The app provides visualization of past journal entries. A study using a mixed-methods approach to evaluate impact and engagement levels among high engagement and low engagement users showed higher improvement on depression metrics for high users compared to low users. The majority of users found the app experience helpful and encouraging [59].

**Woebot:** Woebot is an automated conversational agent designed to deliver CBT and psychoeducation in the form of brief, daily conversations and mood tracking [60]. After gathering responses to self-monitoring data, participants are prompted to learn more about concepts related to CBT by means of video or text. Weekly graphs are used to provide users with weekly mood descriptions for pattern recognition. The chatbot uses empathetic responses, prompts users to interact with the app, and tailors content based on their mood state [60]. An initial study showed that after two weeks people who spoke to Woebot felt better than those in the comparison group [60].

### 1.1 Data collection

This research employed remote methods for data collection as a way of accommodating the needs and challenges faced by people using self-management technologies to support anxiety management. The remote methods used were the use of daily online questionnaires hosted on the Qualitrics platform and optional online semi-structured interviews conducted on Zoom at the end of the study. The main reason for choosing methods that do not require in-person attendance was to cause minimum disruption to the daily lives of participants and lower participant burden. In addition, recruited participants awaited support for treatment and were not yet attending in-person counseling services. Data collection and communication took place individually for each participant. The study took place in Ireland and was approved by the SCSS Research Ethics Committee (REC) at Trinity College of Dublin.

### 1.2 Eligibility criteria

A participant was considered eligible for recruitment if they were between 18–30 years old, proficient in English, and willing to use a mobile app to support their mental health in relation to anxiety management. Additionally, participants were eligible to participate if they owned a smartphone (Android running version of 4.1 or later, iOS version of 10 or later), and had internet access. Participants who contact the SCS for support are offered an initial 45-minute assessment appointment with a member of the counselling team to determine the most appropriate support needs. Students who are deemed high risk (at risk of harm to self, at risk of psychosis or other mental health crisis) are assigned top priority and are seen as soon as possible. The purpose of the study is to improve the design of mobile apps targeted for that purpose and did not involve assessment of the mental health of participants. Therefore, we recruited all participants that met the inclusion criteria. We recruited participants currently on the waiting list to receive treatment at a university counselling service. The purpose of the study is to inform the design of mobile apps targeted for that purpose, and did not involve assessment of the mental health of participants. Therefore, we recruited all participants that met the inclusion criteria. However, it can be assumed, given the assessment procedure, that those participants who were invited to participate in the study had mild to moderate mental health difficulties. In addition, participants who voluntarily withdrew from our study remained on the waitlist to receive support from the service.

### 1.3 Procedure

The Student Counselling Service (SCS) of the university emailed an invitation (with a web link to the study’s Participant Information Leaflet, and Informed Consent Form) to all clients on the SCS waiting List. Additional emails were sent to new clients over time until recruitment was complete. The invitation provided the names of the three apps recommended, and the option to access the informed consent procedure where participants gave the researcher’s permission to email them the surveys. Upon giving consent, we emailed participants selected for inclusion in our study information about the start date of the study and the procedures that would follow. An app information sheet was available to the participants the day before the study started for them to decide which app/s they would be willing to use. We asked participants to read carefully the description page of each app on the marketplace before deciding which app/s to use for the study. The app information sheet is available in S2 Appendix. An email was sent to all participants at the start date of the study reminding them to download and start using the selected app/s.

**Fig 1:**
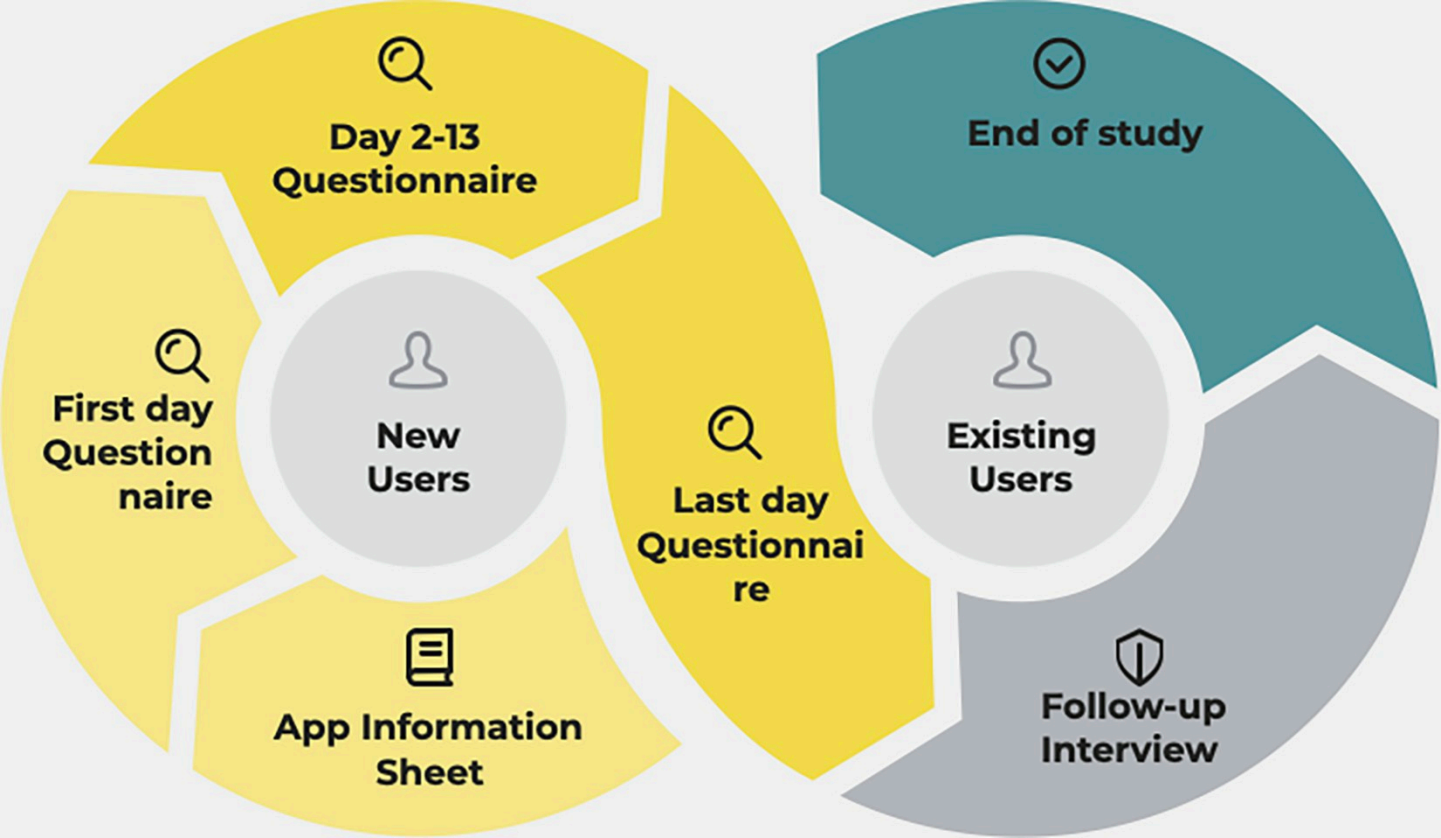
Study process.

All participants were then asked to use up to two of the suggested apps at least once per day for a period of two weeks. We provided users with this option to understand the reasons for app selection and allow for the fact that participants might in reality try a number of apps. Previous research has shown that highly-rated apps tend to be downloaded more frequently than those with lower ratings [22,26]. All apps were free to download and two of them offered in-app purchases. Participants were requested to use the free version of the apps but had the possibility to use the premium version at their own expense. At the end of each day, participants were sent emails reminding them to complete an online questionnaire concerning feedback about the usage of different app features. One of the researchers (AB), would check the questionnaire responses on a daily basis. Participants who did not respond in more than two days in a row, received an email reminding them about the study process. All participants were invited to an optional online semi-structured interview after the end of the two week period. The aim of the semi-structured interview was to uncover in-depth the participants’ experience of using the mobile applications. We conducted interviews that lasted about 30 minutes and were recorded and transcribed by one author. Topics during the interviews comprised features users liked/disliked, suggestions for improving the existing apps, and overall positive and negative aspects of users’ experiences with the app/s over the period of two weeks. The survey items and interview questions are available in S3 Appendix. Fig 1 shows the study process.

Procedures were in place to ensure the safety of the participants. If a participant were to experience discomfort during the study, as students of the university participants were eligible to avail of free support from the Student Counselling Service (SCS). Additionally, standard out-of-hours supports normally advertised to SCS clients were discussed at debriefing. This list of support services was provided at the end of each survey daily and included contact information and direct links to the Student Counselling Service (SCS) and different crisis mental health services across Ireland. The same procedures were followed for participants who participated in an individual online interview at the end of the study period. The user study was conducted in English and data collection took place between June and August 2021. The initial recruitment started in June; an additional email was sent a month later to all clients on the waitlist to receive treatment in order to complete recruitment. All participants received a 40€ voucher after the end of the study.

### 1.4 Measures

The daily questionnaire took about 5–10 minutes to complete and participants provided responses to a set of questions related to the different functionality types, and time spent using the application daily. In addition, the first-day questionnaire included general questions regarding the interface design of the app, and the last day questionnaire included questions related to the usage of app features during the two week period, and the User Version of the Mobile Application Rating Scale (uMARS) [61]. uMARS provides a 20-item multidimensional measure of an app’s quality by assessing the dimensions of engagement, functionality, aesthetics, information, and subjective quality on 5-point scales. The subjective quality dimension is based on four questions: willingness to recommend the app, anticipated app usage frequency, willingness to pay for the app, and overall rating. One further subscale, consisting of 6 items is used to measure users’ perceived impact of the evaluated app on the users’ knowledge, attitudes, and intentions related to the target health behaviour (managing anxiety). A total uMARS score is calculated by averaging all subdomains, whereas subjective quality is calculated by averaging its related subitems or reporting individual items.

### 1.5 Data analysis

We used descriptive statistics to analyze the results from the uMARS questionnaires. We performed qualitative analyses on the participants’ feedback on what they liked and disliked on each day, and on the follow-up interviews following a general inductive approach [62]. One author engaged in close reading of responses and developed initial categories through inductive coding. Coding was discussed with other authors to allow for better familiarization with the data and reduce potential for bias. The authors then agreed upon a final coding framework to be applied to the data.

## 2. Results

Below, we present findings from our qualitative analysis. Semi-structured interviews were conducted to capture richer information and more details about users’ perspectives on the selected apps used during the study and were analyzed separately from the questionnaire data. However, there was a high level of overlap in the analysis among those. Therefore, we present the combined results relating to the same topics and representing the views and experiences of users. More specifically, we merged results for topics concerning participants’ reasons for using different app features, reasons for and barriers to engagement with the apps, as well as suggestions for improvement. We present the results by referring to participants’ comments using a unique number for each identified statement and distinguishing comments derived from interviews (I) and questionnaires (Q).

### 2.1 Participants

Seventeen participants completed the study and eleven participants participated in the optional semi-structured follow-up interviews after the end of the study. Participants completed a mean of 10.5 questionnaires (SD = 2,37) during the two-week study period. Participants were young adults ranging in age from 18 to 30 with a mean of 24.17 years old and were mostly female. App selection varied among participants and most of them decided to use only one app during the study period (Table 1).

**Table 1:** Demographic characteristics & App selection of study participants.

Attribute	Range	Sample size
Gender	Female	14
	Male	2
	Non-binary/ third gender	1
Age	18–25	10
	26–30	7
Education level	Leaving Certificate	6
	Bachelor’s degree (e.g. BA, BS)	6
	Master’s degree (e.g. MA, MS, MEd)	5
	Doctorate or professional degree (e.g. MD, DDS, PhD)	1
App selection	Sanvello	4
	Wysa	3
	Woebot	5
	Sanvello & Wysa	3
	Sanvello & Woebot	2

### 2.2 Reasons for selecting apps

Participants installed apps that seemed to be more suited to their needs (7/17) or based on satisfaction with app branding elements such as logos, app name, and app screenshots (6/17). Four participants installed apps based on consumer reports data such as positive reviews (3/17), and high rating scores (1/17). Other participants mentioned installing apps because they offered a variety of content (3/17), the option to connect with a therapist in the premium version (1/17), or based on the ordering of the options (1/17). One participant decided to try two different type of apps (an app that integrated an automated conversational agent and one that did not) (1/17).

Participants were informed before and during the study that they should download and use up to two apps. One of the participants (P11) decided to download a second app on the third day of the study to find specific functions not included in the initial app choice (i.e. screening, self-monitoring, and information on a specific issue). Another participant (P14) downloaded and used all three apps for the first two days before making a final decision. The participant discontinued use of one of the apps because he was not satisfied with chatbot conversations. Similarly, P3 downloaded two apps on the first day of the study but immediately stopped using one of them when it mentioned a paid membership.

### 2.3 App use

#### 2.3.1 Average time spent

The average time spent using the apps daily differed among participants. The majority of them reported spending an average of 0–15 minutes daily with the app/s. Only three participants reported consistent regular average time spent. Two of them spent a daily time range of 0–15 minutes, and another spent approximately 30 minutes.

#### 2.3.2 Daily reminders for app use

Participants used different strategies that reminded them to interact with the app/s daily (Table 2). Most of them used a combination of approaches to interact with the apps such as receiving reminders from the app (8/11), receiving email reminders from the research team (5/11), doing it in response to events or sensations (e.g. to stop a panic attack or reduce anxiety) (8/11), receiving notifications when they did not use the app for a while (3/11), doing it automatically (3/11), or by setting their own reminders. Six participants interacted with the apps because they received reminders from the app (4/6), or they received an email from the research team (3/6). Five of them mentioned that close to the end of the first week, they continued interacting with the app/s because it became part of their daily routine.

**Table 2:** Participants reminders for app use.

Participant	Receive reminders from the app	Have my own reminders (e.g. in a separate reminder app)	Receive notifications when I don’t use the app for a while	It’s part of my daily routine	I do it automat- ically	I do it in response to events or sen- sations (e.g. to stop a panic attach or to reduce anxiety)
P1	✓				✓	✓
P2	✓		✓			
P3						
P4	✓	✓	✓			✓
P5	✓				✓	
P6	✓			✓		✓
P7				✓		✓
P8						✓
P9	✓					
P10	✓					
P11				✓	✓	✓
P12	✓			✓		✓
P13					✓	
P14	✓					
P15	✓		✓	✓		✓
P16	✓					
P17	✓					

#### 2.3.3 Interaction and use of app features

**Fig 2:**
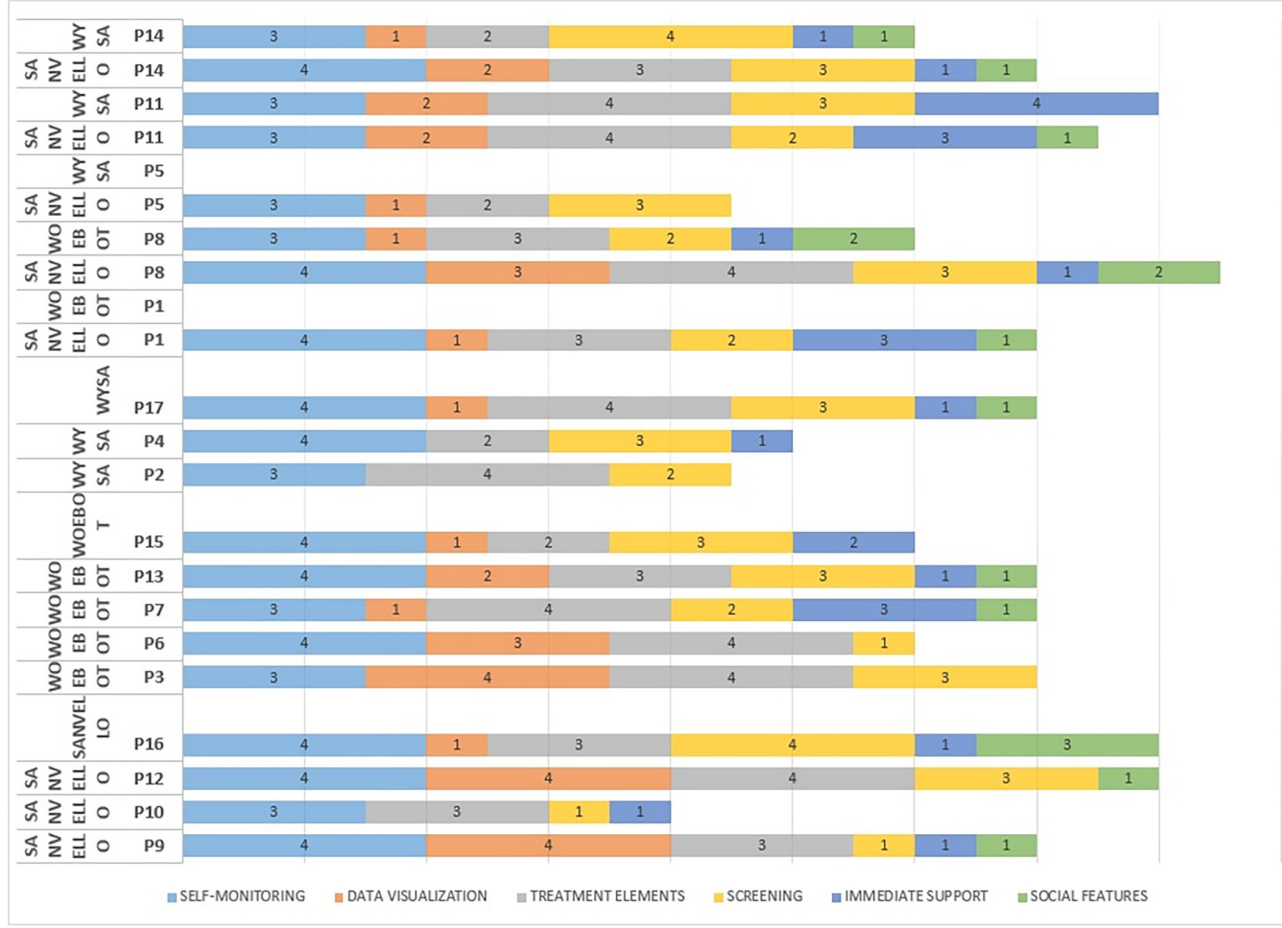
Features used most and least frequently.

The results showed that there is variability in the functions that participants have used among the same apps during the study period. Self-monitoring and treatment elements seem to be the most used functions among all participants (See Fig 2).

The main reasons for participants to use different app features included an interest to improve their mental state concerning anxiety or low mood symptoms (16/17), self-monitor their mood (12/16), use the features based on app recommendations (7/17), or to learn and try new tools to manage their mental health (7/17). Other reasons reported included an interest in reflecting on the collected data (4/17), to make progress (3/17), or due to an occurring crisis/panic attack (2/17). Six participants reported using an app based on reminder emails (2/17), for no particular reason (2/17), and in order to complete the study (2/17). One participant mentioned using the app one of the days because he felt lonely and had no one to talk to and share his thoughts. Table 3 summarizes user motives to interact with different app features.

**Table 3:** Reasons for using app features.

App Features	Participants (N = 17)	Reasons for use	Reasons for non-use
Data sharing	5	willingness to share issues	N/A
		explain app benefits	
Customization	8	interface (select a different backgound, set a nickname)	N/A
		data visualization	
		treatment elements (e.g. alter background sound for meditation exercises)	
Discussion groups	6	check activity for specific groups of interest	no activity on the groups
		read other people’s stories and compare to themselves	
		share something with the group	
		share something with the group rather with the family	
Premium version	11	motivation to use the app	high-subscription costs
			inability to cover app costs through health insurance
			limited free content
			repetitive content
			restricted access to other app features
			satisfaction with content offered in the free version
			no need of additional support
Gamification	9	track progress	inability to progress in the free version
		sense of accomplishment	little effort required for that purpose
		felt rewarding	

#### 2.4 Engagement over time and barriers preventing sustained use

**Fig 3:**
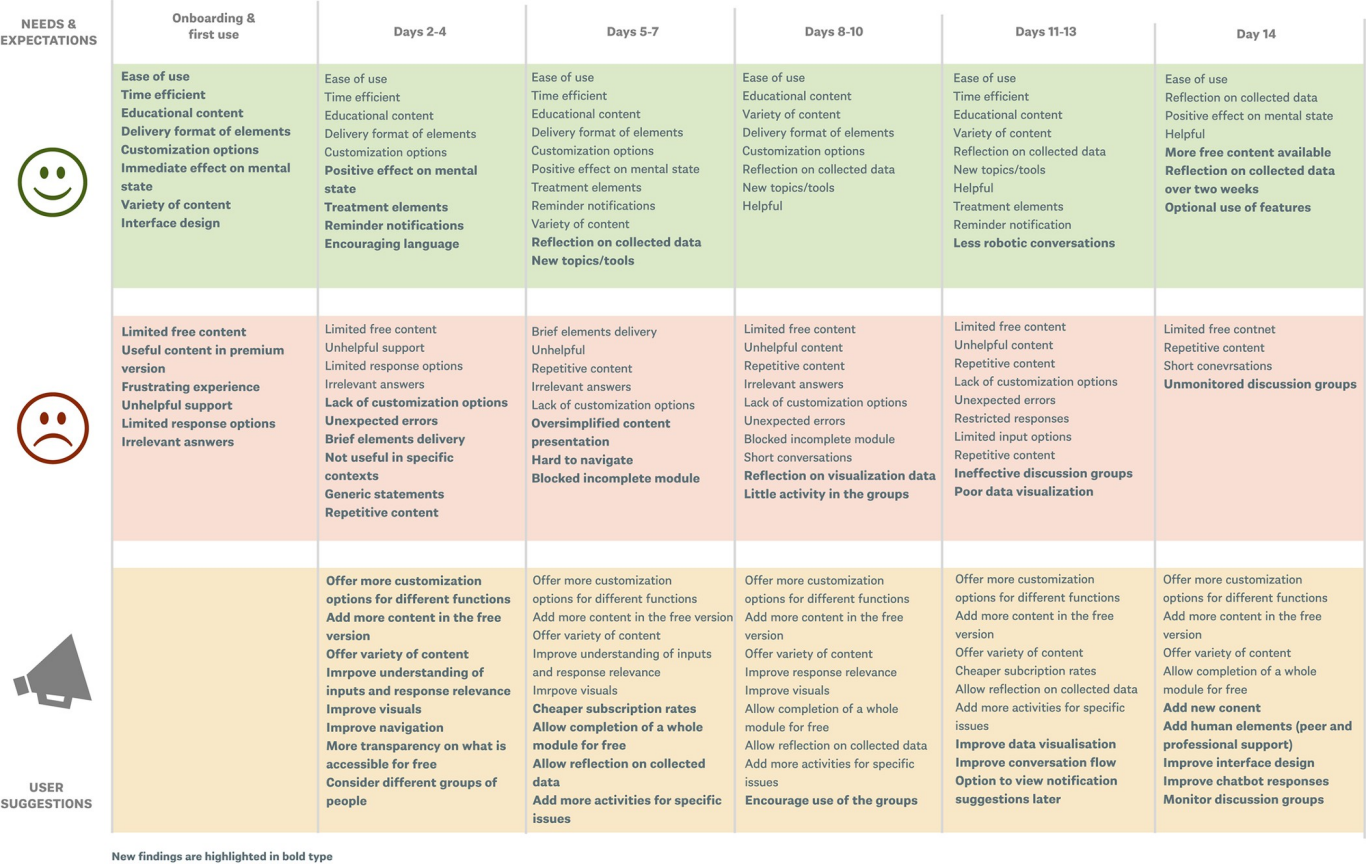
Experience Map.

The results identified several reasons and barriers to engagement, as well as suggestions for improvements for the existing apps to different time points in real-world settings (See Fig 3). Participants’ impressions during the first days of use strongly influenced opinions about the apps, which remained consistent during the second week of the study. The following section describes participants’ experiences with the apps at different time points.

#### 2.4.1 Onboarding and first use

Participants described both their positive and negative experiences during the first day of use. Participants valued apps that are easy to use, provide clear information, offer an aesthetically pleasing interface, and variety of content that is educational. As P17(Q) stated, “I liked the interface and the various options on the app”. Additionally, one participant noticed an immediate effect on his mental state. P4(Q) “I’ve been feeling super anxious with [a personal event] coming up, so I wanted to check in to see what my needs were and then do some guided mediation to de-stress” and “It calmed me down”. Participants mentioned satisfaction with apps that can be used in a time efficient manner and provide several customization options. For example, P17(Q) liked “The interface and the various options on the app.”, and P3(Q) mentioned that the app “was very easy to use and very time-efficient”.

Conversely, two participants experienced a confusing experience during their first-time use. For example, P11(Q) stated, “when I first downloaded it I felt slightly overwhelmed by all the content”. Participants reported that a variety of content or useful content is not available in the free version of the apps. As P12(Q) stated, “I wish more courses or mediation sounds were available without a subscription”, and P2(Q) “Most of the useful content is only accessible if you pay”. Additionally, participants were provided with limited response options during their interaction with a chatbot. As P6(Q) stated, “A lot of the replies you give have to be chosen from a preselected list”. Another participant mentioned receiving irrelevant content based on their inputs while interacting with the app. As P13(Q) stated, “the insight didn’t really pertain to the mental health problem I brought up”.

#### 2.4.2 Week 1

Over the following days, participants reported additional positive and negative experiences with the apps. The apps helped participants to learn new tools, provide a sense of achievement, and can have a positive effect on participants’ mental health state. For example, P10(Q) stated, “I was able to check off some goals. Felt a sense of achievement”, and P11(Q) “I enjoyed learning how to reframe my thoughts as well as the examples of the 10 thinking traps.” Participants value the reminder notifications, and different treatment elements offered in the apps. For example, P11(Q) stated, “I like using the reframing CBT component as I like to be able to view my thoughts in a different way”, and P7(Q) “It is a fast piece of work and reminds me to check in on myself even though I am feeling ok today.” In addition, participants value the encouragement language used to deliver different treatment elements. As P6(Q) stated, “The AI offered very kind words. It’s hard at first to take seriously words of encouragement from a scripted AI but I think the benefit is more about telling yourself those words”. Additionally, participants described liking the delivery format of different elements. For example, P3(Q) described “Very relaxing visuals”, and P5 stated “There were both videos and reading exercises to complete which made it less tedious”. Towards the end of the first week, participants appreciated the new content provided and the ability to reflect on the data collected through the apps. As P12(Q) reported, “I liked seeing the progress I have made mood wise”, and P9(Q) “was able to track my mood and see how it compared to other days”. During the first days of use, participants disliked receiving repetitive content and generic statements based on their inputs. For example, P16(Q) stated, “I feel as if the app may become a tad too predictable. I would prefer if it felt less repetitive but with an app interface as opposed to a human this might be hard to avoid”. Other participants were dissatisfied with the content delivery and found the content delivered to them to be irrelevant. As P10(Q) stated, “The example given about having a fear of spoons was a bit ridiculous. Perhaps they were trying to be whimsical but it would have been more meaningful if they gave a more realistic example..”, and P11(Q) “the bot may be referring to the past tense when the stressful event was happening at present”. In addition, participants reported mixed feelings about the time required to use the app. For example, P2(Q) stated “Tends to want me to engage longer and I don’t have the time”, and P12 “The lesson for today’s guided journey was very short. It was reading a few slides that took me probably 30 seconds in total”. Some participants disliked the simplified delivery of content, “I found the module elements eg videos and reading to be oversimplified” P14(Q). Participants mentioned the lack of customization options for different app features such as interface, reminders, and different treatment elements. For example, P3(Q) stated, “I wish I could pause session and continue later”, and P10(Q) “I was unable to add multiple reminders”. Other participants’ experience was interrupted unexpectedly during app use caused by functionality issues such as broken functionality of app features. For example, P12(Q) stated, “I tried the 10-minute meditation and found that after a couple of minutes the sound would randomly turn off and I would have to go in, pause the music, then play it again for it to come back. It interrupted my meditation”, and P2(Q) “I was really stressed, I told the app that, it told me to breathe and then the app stopped responding. There was no way of resuming the conversation and I could have done with it’s help”. Additionally, certain treatment elements require participants to use an app in their home environment, preventing them from using it in different settings. As P3(Q) stated, “Asked me to work on my sense of smell but I was in public and wearing a mask but I couldn’t go back in the app so I had to click through the smelling exercise”.

Towards the end of the first week, participants also experienced problems with app navigation. For example, P11(Q) stated, “I found it difficult to remember where to find the specific progressive muscle relaxation exercise I had completed previously. This exercise was part of the guided journey so I had to back through each completed lesson to find it”. In addition, participants’ experience was interrupted during use when premium access was required to complete modules. As P10(Q) stated, “I was stopped from completing the module I was working through this week. Premium access is needed for the last two tools in the module.”

#### 2.4.3 Week 2

There are a small number of new positive and negative experiences that participants reported during the second week of the study. P14(Q) reported that the app becomes more responsive and conversations feel less repetive after a few days of use, “I actually feel like the longer I’ve used wysa the more responsive it seems so i was happy with that”. P16(Q) reflected on app’s positive effect on the mental state, “Today I really recognised the value of the app. It has had a positive effect on my mental health over the past two weeks”. Other participants valued the reflection on the collected data over the the two week study period. As P6(Q) stated, “Reviewing my mood graph over the past 2 weeks. My prevailing mood seems to be content or happy with some anxious periods that can last 2 or 3 days. The app in this case helped me realise that even though sometimes it feels like I’m never happy, the truth is I usually am but maybe I’m just going through a rough couple days.”

Collection of user data and reflection on data visualization can have an adverse affect for other users. As P9(Q) stated, “I disliked seeing how shit my mental health has been over the past few days”. In addition, the collected data visualized should provide value for the users. As P2(Q) stated, “I wanted to check my progress for the past week but it just listed everything that I had inputed. Wasn’t very helpful”. Participants described little activity in the group feature of the apps and lack of moderation of activity. As P12(Q) stated “I was upset because the groups I was interested in didn’t have any activity for the past couple of months, so I decided to leave them”.

#### 2.4.4 Suggestions for improvement

Participants provided several suggestions for improvement during the first week of use. Many participants referred to cost related issues that affect app use in daily life. Participants requested the availability of more content, and the ability to complete interacting with app features in the free version, rather than being blocked halfway through. For example, P10(Q) stated, “Allow for completion of a complete module and reserve other complete modules for premium.”, and P17(Q) “Add more features to the basic subscription even if it’s for a trial period first”. In addition, participants requested cheaper subscription rates for specific types of users (i.e. students) and the possibility to access subscription plans through their healthcare insurance. For example, P11(Q) stated, “Have more content available without subscribing to premium, have a student premium subscription rate..”. Other participants requested more transparency on the content that is accessible in the free version. For example, P11(Q) stated, “The exercises I was recommended all required premium when I clicked into them. I would like a breakdown of what content can be accessed without premium and what content is accessed only with premium”, and P2(Q) “Less restricted features in the free version. Better transparency of what is/is not accessible through the free version. Tracking of time spent on the app/progress.”

The majority of participants requested more customization options for different functions of the app. Participants wanted to be offered more options to alter the interface, the reminders, and the treatment elements on the apps. Participants wanted to customize the visual aspects of the interface such as to change the background colors or background music. For example, P16(Q) requested changing “the intense colours of the interface”. In addition, they requested better navigation during app use. As P13(I) stated, “I want the ability to take a break from topics easier”. Other participants requested the ability to set multiple reminders, and the option to view notification suggestions later. As P2(Q) stated, “More freely accessible materials, if you receive a notification from the app offering a suggestion, allow me to view the suggestion later in my app. I cannot find most of the exercises it suggests later on when I have time to do them”, and P10(Q) requested the “Ability to add multiple reminders”. Participants requested more customization options for the treatment elements of the apps. Such customization options for example should allow to alter the delivery format of content. For example P7(Q) stated, “Audio options for the breathing exercises, a feature to track your mood/symptoms each day when you log on” and add the option to voice record the responses, and P4(Q) requested “less text oriented, more options for meditations”. Other users requested more options to enhance the experience with the treatment elements such as to change the speed of a meditation or to add a pause and continue button for this purpose. For example P11(Q) stated, “The audio clips could have the ability to fast forward and rewind”. Participants wanted to be able to use the apps in different contexts (add video subtitles, continued sound looping). As P7(Q) stated, “Option to give feedback on a scale rather than just negative or positive, subtitles for videos so they can be watched quietly”. Other participants requested the ability to customize the length of their interaction with different app features by offering shorter or longer app sessions to suit users’ unique needs. For example, P2(Q) requested, “Shorter conversations, less content restricted to Premium, track progress”. Participants wanted an enhanced experience with the chatbot conversations. Conversational interfaces should better understand users’ inputs and provide more relevant content to them. As P2(Q) stated, “Better understanding of whats imputed or more multiple choice responses”. Participants requested more activities for specific type of issues or target user groups. For example, P6(Q) stated, “Maybe expand to include some LGBT supports, and also eating disorders”, and P11(Q) “More articles on general anxiety related issues or mental health issues ie. Relationship difficulties, grief, addiction etc.”.

During the second week, participants discussed the need to receive new content that is non-repetitive. As P16(Q) stated, “To have a greater pool of content and responses from the bot so the experience feels fresher”. P11(Q) requested customization of the interface to have easier access to features of their preference, “Have the suggested exercises/ options at the top of the home page. Have less of the home page taken up by unesscessary/ not directly relevant information”. P12 wanted apps to encourage the use of the groups and P14 the ability to monitor chat groups. As P12(Q) stated, “somehow find a way to encourage more use of the groups”, and P14(Q), “I really strongly disliked the chats, they should be monitored so not to enhance individuals issues as people are likely opening them during moments of vulnerability”.

### 2.5 Overall engagement

Participants during the interviews described their overall motivation to use the apps during the two weeks. Five participants mentioned that their motivation to use the apps declined over time. Reasons for that included repetitive content, inability to complete a journey, lack of visual appeal in certain features, and an unimproved mental state.

Four participants reported pretty consistent motivation to use the apps. For example, P15(I) stated, “I probably used it between 10 and 15 minutes a day regularly but there were 2 or 3 days where I either used it a little bit longer or I went back to it and used it again a second time that day so my engagement was pretty steady with it most days”. Another participant was less inclined to use the app towards the end of the first week because of the lack of options but stated that at the start of the second week it became part of his daily routine. As P12(I) stated, “I think the first few days I was definitely the most intrigued to just kind figuring out like what the app was all about, what I can access, how beneficial would be.. my interest kind of renewed a bit then at the start of the second week kind of like OK cause now it is part of my routine and after work I would be checking with the app and then it just kind of felt instinctual so then it was not necessarily, it was just kind of time to do it now”. Only one participant mentioned an increased motivation to use the app based on data reflection. As P9(I) stated, “I would say the more I have used it if that makes sense so I was using it more because there were more data for me to look at and you know become more comfortable with the app.”

Participants also referred to their motivation to use the app in their daily life. Eight participants mentioned that they would have used the apps less frequently outside of a research setting. A few of them reported using the apps on specific occasions during stressful days. For example, P12(I) stated, “I would probably not use it as a daily type of thing but I think if I am having an issue with anxiety that day I probably will check in and check if there is a tool that I could use like meditation or mindfulness that day but I do not think it will be a daily routine”. Only one of the participants mentioned daily app use during and following the end of the study. As P4(I) stated, “I’ve actually continued using it just because I think it was a good way for me to keep track of how I’ve been doing and feeling. And so I really enjoyed that. And I kind of got in the habit of doing it over the time that I was doing it.”

### 2.6 Continued use

Despite the identified barriers to engagement, nine participants were interested in using the apps after the end of the study period. Only two participants reported unwillingness to use the apps after the end of the study, because the free version limits access to app features and restricts completion of modules. As P2 (I) stated, “I would think of using it for the more general things, but the general things that are in the free version are very, very limited.”, and P10(I) stated, “And like I said, the thing that they know people with anxiety and OCD and who are stressed out like one of the things that they know affects them, like, you know, feeling like you’re always in the middle of things and not get in a sense of completion or accomplishment or progress or whatever.”

Two of the participants that used the same set of two apps indicated different preferences among them for future use. As P14(I) stated, “I think Wysa is more engaging, I suppose when you attempt to direct the conversation you know sometimes it may not go with you but Sanvello is more like finding what you are looking for and then I was really disappointed with the chat/group discussions I found so I would definitely not use Sanvello then based on that.”

In contrast, P11(I) considers the chatbot app time-consuming and indicated a preference to use only a specific feature on this app compared to the other app. “I was just drawn to Sanvello app more. I find it more user-friendly, I think again the Wysa is very focused on kind of self-talking and CBT which those require a lot of time, and to be able to have a conversation it requires 10 minutes or more.”

### 2.7 App quality rating

Overall, the results of the uMARS questionnaire suggested that the 3 apps were of moderately good quality [61]. We removed a single uMARS questionnaire from one participant who used multiple apps without clarifying which one was used. Functionality was the domain with the highest rating, followed by information, aesthetics and engagement (See Table 4). Subjective quality scores were generally high. Most users would use the apps in the future, consider recommending the apps to people, and provided an average or high overall rating for the apps (See Table 5). Even though the apps were considered highly functional and received good subjective quality scores, users were not willing to pay for the apps, in line with the results of the daily questionnaires and interviews. Perceived impact scores showed that for most of the participants, apps were felt to have increased intentions/motivation to manage anxiety, encouraged them to seek further help, and decreased anxiety symptoms.

**Table 4:** User version of the Mobile App Rating Scale scores.

Scores	Sanvello	Wysa	Woebot	Total Average Mean Score
Engagement Mean Score	3.82	3.5	3.47	3.59
Functionality Mean Score	4.46	4.04	4.33	4.27
Aesthetics Mean Score	4.47	3.99	4.1	4.18
Information Quality Mean Score	4.39	4.16	4.15	4.23
App Quality Total Mean Score	4.28	3.92	4.01	4.07

**Table 5:** Subjective quality scorers of the uMARS questionnaire.

Subjective Quality	Sanvello	Wysa	Woebot	Total Average Mean Score
Would you recommend this app to people who might benefit from it?	4.14	3.33	3.66	3.71
How many times do you think you would use this app in the next 12 months if it was rel- evant to you?	3.71	2.83	3.5	3.34
Would you pay for this app?	2.57	2	1.5	2.02
What is your overall (star) rating of the app?	3.71	3.66	3.5	3.62
App Subjective Quality Total Mean Score	3.53	3.12	3.04	3.23

## 3 Discussion

### 3.1 Principal Findings

The current study offers the opportunity to understand how apps are used in real-world settings and the reasons affecting user interactions over time. This work contributes to a deeper understanding of user engagement with mobile apps for anxiety on a day-to-day basis. The study also affirms the results of prior work [48–50,63,64], which has identified reasons and barriers to engagement with such apps. Our study has further related the identified barriers and suggestions for improvement to different time points by investigating users’ perspectives in daily life over the period of two weeks.

The results showed that users form opinions about the apps during the first days of app use. During their first interactions with the apps, they identify barriers preventing long-term engagement and app adoption. User opinions remain consistent even after subsequent app interactions. In line with the results of a previous study [65], we found that users value the ability to self-reflect on the collected data, learn and practice new skills, and make improvements to their health with the use of such apps.

Users value apps that are easy to use, offer a variety of content, and customization options for different app functionalities. Conversely, participants’ motivation to interact with the apps is affected negatively by high subscription costs, limited free content, and a lack of customization options. Participants familiarize themselves with an app’s content quite quickly; content that remains unchanged after several interactions and affects the long-term use of such apps. Echoing [66], there is a need for mental health app developers and evaluators to better understand what leads to sustained engagement, and how to create products that achieve it.

### 3.2 Integrate apps into a system of health care

Mental health apps offer the possibility to provide instantaneous access to help in moments of need and have the potential to enhance care. Smartphone applications could enhance care for people on the long waitlists to in-person appointments with clinicians by offering low-intensity intervention content when in need of support. An interesting aspect of the study is the way that some participants used multiple apps, either by moving to a new app (abandoning the first), or used multiple apps simultaneously. Understanding use of apps in the wild may thus involve understanding user interactions with a set of apps. Similarly, in a study that explored app-use patterns across a mental health app platform with 14 apps, participants seemed to settle on their preferred set of apps from a larger subset [67]. Future studies should explore whether app usage is affected by the choices provided to the users on the waitlist to receive treatment and if single app use affects engagement. User impressions form fairly quickly, and additional support in the early stages of app adoption could help them identify apps relevant to their needs and enhance app use.

Another possible solution to enhance care could be the use of smartphone mental health apps in conjunction with therapy and to blend smartphone apps and offline tools. For example, a new ecosystem integrating apps and online resources into existing care pathways, provided via clinician referral and self-care pathways, expanded a health system’s ability to care for patients during the Covid-19 pandemic [68]. Integrating digital tools in this way increased the uptake and engagement with these tools [68]. The exploration of such services should involve collaboration with clinicians and service users to provide content relevant to clients and rethink app design by addressing user needs, and consider the setting of delivery, for example users that are on the waitlist to receive counseling.

### 3.3 Understanding individuality and addressing diverse user needs

App content is not targeted to suit participants’ unique interests and needs. Similar to the results of previous research [22,26], a few users in our study relied on the app description and reviews to assess their usefulness; descriptions that may not be representative of an app’s actual functionality. Users and providers need reliable resources to determine which apps may be most helpful for specific group of users. Therefore, several evaluation guidelines exist to guide users through a number of questions to decide whether or not to proceed with using an app [69,70]. In addition, independent app rating platforms offer an important service and help consumers and clinicians distinguish high-quality apps [71].

The results indicate that individual differences can influence an app’s uptake. We observed a variety of styles of use and preferences among app features with self-monitoring and treatment element features being the most used. In addition, we observed that users’ time spent interacting with the app differs. Future studies should explore such differences by investigating the design of mental health apps with a specific type of user. The variation in preferences for intervention content and features used among users indicates that mental health app designers should explore broader requirements when designing apps such as demographics, cultural background, and the diversity of mental health needs among users of such apps.

The commercial apps used in this study offer a subset of content and features in the free version. Users disengage from commercial apps due to perceived high subscription costs and limited content in the free version of such apps. The business models on which these apps are based require subscriptions or gated features, leading to a degree of conflict between user needs and the need to generate revenue to stay in business. In addition, apps provide fixed content and do not take into consideration long term user interactions. App designers should consider the different contexts that apps are used and different time intervals and interactions of users with such apps. Users’ interaction with a limited amount of free content, and familiarity with unchanging app content, influence app uptake, and adherence. Future apps should be designed by adapting to the long-term needs of users.

Even though participants’ motivation to interact with the apps daily differ, most of them were willing to use the apps after the end of the study. Apps hold the potential to enhance care in daily life but sustained interactions are not well supported. Mental health app designers could design services that are more personalized and engaging by taking into consideration different user characteristics and interactions over time. This might involve refreshing content after a user has used an app for some period of time, and considering at the design stage the potential longer term use of the app. Future studies should explore further users’ interactions with different app features in real-world settings over time to inform the design of engaging apps.

## 4 Limitations

This study has several limitations. Even though participants were on the waitlist to receive counseling and interested in using mobile applications to support their mental wellbeing in relation to anxiety management, we did not take any further measures to determine if participants met the criteria for anxiety disorders. The apps used in this study are not representative of the full range of apps available in the app stores due to the selection criteria of the study (CBT apps that are widely used and present some evidence for effectiveness for anxiety management and engagement metrics) and the fact that most apps in the app store do not have any evidence supporting their effectiveness [19]. Thus, using a different set of apps might reveal different barriers, facilitators, or design considerations. In addition, participants did not have access to the full content within the applications which affects the understanding of user engagement with premium app features and content. As the aim of the study was to understand user perspectives, we did not focus on comparing individual apps. Overall, our approach provided data on real-world user experiences across a set of apps that are available, widely used, and have some evidence to support them.

## 5 Conclusions

Understanding daily perspectives on the usage of mental health apps used for anxiety management is critical for the design of apps that are more likely to be adopted and used for a long period of time. We conducted a user study to understand how such apps are used on a daily basis by young people awaiting treatment, in order to identify barriers to engagement at particular time points, and ways to improve the design of these apps. This study extends the literature by highlighting barriers and factors affecting engagement at different time points. Overall, our results show that initial usage forms and shapes users’ perspectives on these apps, which then remain largely unchanged. Although apps integrate a range of functionalities, many app features are not used often and the same apps are used differently by different people. Furthermore, long-term use is affected by cost-related issues, familiarization with an app’s content, and a lack of personalization options. Future research should explore user engagement and interactions over time with respect to these matters by considering long-term interactions with such apps, to realize their potential to enhance care. Without clear, understandable requirements for the design of anxiety management apps in different contexts and conditions, engagement will remain an ongoing challenge for this type of mental health-related technology. We hope that our results can inform the development of mobile mental health applications for anxiety, and help improve the design of such apps.

## Supporting information

S1 Appendix Functionality of selected apps. (PDF)

S2 Appendix Participant app information sheet. (PDF)

S3 Appendix Survey items and interview questions. (PDF)

S4 Appendix Supporting data. (XLSX)
