# Supplementary material for: Examining young adults daily perspectives on usage of anxiety apps: A user study
Source: PLOS Digit Health. 2023 Jan 26;2(1):e0000185. doi: 10.1371/journal.pdig.0000185 (PMC9931254; doi:10.1371/journal.pdig.0000185)
Supplement: S1 Appendix — (PDF) [file pdig.0000185.s001.pdf]

APP FUNCTIONALITY	SANVELLO	WYSA	WOEBOT
FIRST-TIME UX	Notification setup Account creation	Onboarding screens, Nickname customization	Onboarding screens Privacy policy Optional account creation Nickname customization Notification setup
TAILORING DURING FIRST USE	Select up to 3 goals to work on (8 in total)	Select challenges (16 in total) Option to skip this step	✗
SCREENING	Screening of distress in 3 dimensions	PHQ9 & GAD7	To learn about yourself over time
SELF-MONITORING	Mood	Mood	Mood
SELF-MONITORING TAILORING	5 interventions (1 intervention for each distinct feature)	3-5 different each time	Same interventions based on specific entries
MOMENTARY SUPPORT	Resources	When you type ‘help’, ‘sos’ features with 3 functions, safety plan	When you type ‘sos’ Help me feel better’ feature
DATA VISUALISATION	Self-monitoring entries Data comparison Completed modules	Past conversations with the chatbot	Self-monitoring entries Gratitude & journal entries
REPORTS	Weekly & monthly on the website	✗	After 1 week of use
GAMIFICATION	Level upgrades when completing modules	✗	✗
IN-APP SUPPORT	Discussion & chat groups	Premium therapist support	✗
DATA SHARING	Add a code to allow a therapist view the data, weekly report through email	Data are shared with an expert	✗
PUSH NOTIFICATIONS	Random or specific times selection, on/off notifications for app features	Fixed times, notifications to access intervention strategies	Specific times (night, day, morning, evening)
CHATBOT	✗	✓	✓
SENSORS	✗	✗	✗
TREATMENT PROGRAM	✓	✓	✗
PSYCHOEDUCATION	✓	✗	✓
COGNITIVE TECHNIQUES	✓	✓	✓
RELAXATION	✓	✓	✓
BEHAVIORAL ACTIVATION	✗	✗	✓
