# Supplementary material for: Examining young adults daily perspectives on usage of anxiety apps: A user study
Source: PLOS Digit Health. 2023 Jan 26;2(1):e0000185. doi: 10.1371/journal.pdig.0000185 (PMC9931254; doi:10.1371/journal.pdig.0000185)
Supplement: S2 Appendix — (PDF) [file pdig.0000185.s002.pdf]

App Information Sheet

The following table contains the list of apps available to download for the study.
Please carefully read the description page of the apps available in the stores to decide the app/s you are willing to use during the study period.

Please do not download and start using the app before the start day of your participation is confirmed!

**App name:**	**Wysa**	**Sanvello**	**Woebot**
**App design:**	Automated conversational agent (chatbot)	Mobile App	Automated conversational agent (chatbot)
**Targeted conditions:**	Mood disorders, Stress & Anxiety, Sleep	Mood disorders, Stress & Anxiety	Mood disorders, Stress & Anxiety, Addictions or Substance use, Chronic pain
**Main Therapeutic Elements:**	Cognitive Behavioral Principles, Mindfulness	Cognitive Behavioral Principles, Mindfulness, Gratitude	Cognitive Behavioral Principles, Dialectical Behavioral Therapy, Mindfulness
**Availability:**	Google Play, Apple app store	Google Play, Apple app store, Online	Google Play, Apple app store
**Available for:**	iOS 10.0 or later, Android 4.1 and up	iOS 10.0 or later, Android 5 and up	iOS 11.0 or later, Android 6 and up
**App store link:**	Android	Android	Android
**App store link:**	iOS	iOS	iOS
