# Supplementary material for: Examining young adults daily perspectives on usage of anxiety apps: A user study
Source: PLOS Digit Health. 2023 Jan 26;2(1):e0000185. doi: 10.1371/journal.pdig.0000185 (PMC9931254; doi:10.1371/journal.pdig.0000185)
Supplement: S3 Appendix — (PDF) [file pdig.0000185.s003.pdf]

Online Questionnaires

Online Questionnaire – Day 1

Q1 Which application/s did you install? Please select all that apply

- ☐ Sanvello
- ☐ Wysa
- ☐ Woebot

Q2 Why did you choose to install those applications?

____
____
____
____
____

Q3 When you started using it, what were your first impressions?

____
____
____
____
____

End of Block: General

Q4 Please answer the following questions (App Name)

	Yes	No
Did you like the overall interface of the application?	○	○
Was the font size suitable for your needs?	○	○
Was the font colour suitable for your needs?	○	○
Did you find the application easy to navigate?	○	○
Did you read all the user instructions the app provided?	○	○
Did you find them helpful?	○	○
Was the application easy to use at first?	○	○
Did it get easier to use the more your used it?	○	○
Could you remember how to navigate the application once you returned to it after a break?	○	○

Q7 Did you use the application/s today?

○ Yes

○ No

Q8 Roughly how much time did you spend using the application today?

- ○ 0-15 minutes
- ○ 15-30 minutes
- ○ 30-45 minutes
- ○ 45-60 minutes
- ○ More than an hour

Display This Question:

If Did you use the application/s today? = No

Q9 Why did you not use the application/s today?

____

Display This Question:

If Did you use the application/s today? = No

Q10 Please open the application/s now and use it for a while before filling the rest of the questionnaire

Q11 What app features did you use today? Select all that apply:

- ☐ Mental Health Screening (questionnaires used to exam your emotional health)
- ☐ Self-monitoring (track mood and/or other factors related to your mood)
- ☐ Therapeutic elements (information about mental health, exercises designed to help alleviate anxiety symptoms)
- ☐ Immediate support (on-demand support provided when indicated you are in immediate need of help)
- ☐ Data visualisation (graphs or charts of your mood, comparison of mood related data)

Q12 What are the reasons for using these functions today?

____

____

____

____

____

Q13 Which options did you use to receive content through the app based on your inputs? Please select all that apply:

- ☐ I selected goal/s or challenge/s to work on when launching the app for first time
- ☐ I received recommendations of exercises to practice based on my mood entries
- ☐ I set up the notification timing
- ☐ None of the above

Display This Question:

If Which options did you use to receive content through the app based on your inputs? Please select... = I selected goal/s or challenge/s to work on when launching the app for first time

Or Which options did you use to receive content through the app based on your inputs? Please select... = I received recommendations of exercises to practice based on my mood entries

Or Which options did you use to receive content through the app based on your inputs? Please select... = I set up the notification timing

Q14 Were you satisfied with the content provided to you based on your inputs?

- ◯ Yes
- ◯ No

Display This Question:

If Which options did you use to receive content through the app based on your inputs? Please select... = I selected goal/s or challenge/s to work on when launching the app for first time

Or Which options did you use to receive content through the app based on your inputs? Please select... = I received recommendations of exercises to practice based on my mood entries

Or Which options did you use to receive content through the app based on your inputs? Please select... = I set up the notification timing

Q15 Please explain why the content provided to you based on your inputs was effective/ineffective:

____

____

____

____

____

Q16 Did you use any customisation options provided through the app? Please select all that apply

- ☐ Interface customisation (e.g. option to customize background theme, select nickname etc.)
- ☐ Treatment-oriented customisation (e.g. options to customise different exercises delivered through the app)
- ☐ Data visualisation customisation (e.g. option to compare data, view different visualisations of data entries etc.)
- ☐ I did not customise my app today

Display This Question:

If Did you use any customisation options provided through the app? Please select all that apply = Interface customisation (e.g. option to customize background theme, select nickname etc.)

Or Did you use any customisation options provided through the app? Please select all that apply = Treatment-oriented customisation (e.g. options to customise different exercises delivered through the app)

Or Did you use any customisation options provided through the app? Please select all that apply = Data visualisation customisation (e.g. option to compare data, view different visualisations of data entries etc.)

Q17 What customisation options did you value most and why? Please explain:

____

____

____

____

____

Display This Question:

If Which application/s did you install? Please select all that apply = Wysa

Or Which application/s did you install? Please select all that apply = Sanvello

Q18 Did you consider subscribing to the premium version of the app?

○ Yes

○ No

Display This Question:

If Did you consider subscribing to the premium version of the app? = Yes

Q19 What are the reasons for considering to upgrade your subscription?

____

____

____

____

____

Display This Question:

If Did you consider subscribing to the premium version of the app? = Yes

Q20 If you have already purchased an upgrade, tell us about your experience with the premium version of the app

____

____

____

____

____

Display This Question:

If Which application/s did you install? Please select all that apply = Sanvello

Q21 Did you use the discussion or chat groups today?

- ◯ Yes
- ◯ No

Display This Question:

If Did you use the discussion or chat groups today? = Yes

Q22 What are the reasons for joining the discussions and/or chat groups today?

____

Display This Question:

If Did you use the discussion or chat groups today? = Yes

Q23 How was your experience with the discussion and/or chat groups?

____

Display This Question:

If Which application/s did you install? Please select all that apply = Sanvello

Q24 Did you complete any modules today?

- ◯ Yes
- ◯ No

Display This Question:

If Did you complete any modules today? = Yes

Q25 A user upgrades level by completing modules. How do you find the idea of upgrading levels after completing modules?

____

Display This Question:

If Which application/s did you install? Please select all that apply = Wysa

Or Which application/s did you install? Please select all that apply = Sanvello

Q26 Did you share with anyone any information you recorded in the app?

- ◯ Yes
- ◯ No

Display This Question:

If Did you share with anyone any information you recorded in the app? = Yes

Q27 With whom do you/did you share the information you recorded through the app? (select all that apply)

- ▢ With my friends
- ▢ With family
- ▢ With my partner
- ▢ Other

Display This Question:

If With whom do you/did you share the information you recorded through the app? (select all that apply) = Other

Q28 Please specify your answer:

____

Q29 How do you remember to use the app? (select all that apply)

- ○ I receive reminders from the app
- ○ I have my own reminders (e.g. in a separate reminder app)
- ○ I receive notifications when I don't use the app for a while
- ○ It's part of my daily routine
- ○ I do it automatically
- ○ I do it in response to events or sensations (e.g. to stop a panic attack or reduce anxiety)
- ○ I receive an email reminder from the research team
- ○ Other

Q30 What did you like most about your interactions with the app/s today?

____

Q31 What did you dislike about your interactions with the app/s today?

____

Online Questionnaire - Days 2-13

Q1 Which application/s did you download to use? Please select all that apply

- ☐ Sanvello
- ☐ Wysa
- ☐ Woebot

Q2 Did you use the application/s today?

- ○ Yes
- ○ No

Display This Question:

If Did you use the application/s today? = No

Q3 Why did you not use the application/s today?

____

Display This Question:

If Did you use the application/s today? = No

Q4 Please open the application/s now and use it for a while before filling the rest of the questionnaire

Display This Question:

If Did you use the application/s today? = Yes

Q5 Roughly how much time did you spend using the application today?

- ○ 0-15 minutes
- ○ 15-30 minutes
- ○ 30-45 minutes
- ○ 45-60 minutes
- ○ More than an hour

Q6 What app features did you use today? Select all that apply:

- ☐ Mental Health Screening (questionnaires used to exam your emotional health)
- ☐ Self-monitoring (track mood and/or other factors related to your mood)
- ☐ Therapeutic elements (information about mental health, exercises designed to help alleviate anxiety symptoms)
- ☐ Immediate support (on-demand support provided when indicated you are in immediate need of help)
- ☐ Data visualisation (graphs or charts of your mood, comparison of mood related data)

Q7 What are the reasons for using these functions today?

____

____

____

____

____

Q8 Which options did you use to receive content through the app based on your inputs? Please select all that apply:

- ☐ I selected goal/s or challenge/s to work on when launching the app for first time
- ☐ I received recommendations of exercises to practice based on my mood entries
- ☐ I set up the notification timing
- ☐ None of the above

Display This Question:

If Which options did you use to receive content through the app based on your inputs? Please select... = I selected goal/s or challenge/s to work on when launching the app for first time

Or Which options did you use to receive content through the app based on your inputs? Please select... = I received recommendations of exercises to practice based on my mood entries

Or Which options did you use to receive content through the app based on your inputs? Please select... = I set up the notification timing

Q9 Were you satisfied with the content provided to you based on your inputs?

- ○ Yes
- ○ No

Display This Question:

If Which options did you use to receive content through the app based on your inputs? Please select... = I selected goal/s or challenge/s to work on when launching the app for first time

Or Which options did you use to receive content through the app based on your inputs? Please select... = I received recommendations of exercises to practice based on my mood entries

Or Which options did you use to receive content through the app based on your inputs? Please select... = I set up the notification timing

Q10 Please explain why the content provided to you based on your inputs was effective/ineffective:

____

____

____

____

____

Q11 Did you use any customisation options provided through the app? Please select all that apply

▢ Interface customisation (e.g. option to customize background theme, select nickname etc.)

▢ Treatment-oriented customisation (e.g. options to customise different exercises delivered through the app)

▢ Data visualisation customisation (e.g. option to compare data, view different visualisations of data entries etc.)

▢ I did not customise my app today

Display This Question:

If Did you use any customisation options provided through the app? Please select all that apply = Interface customisation (e.g. option to customize background theme, select nickname etc.)

Or Did you use any customisation options provided through the app? Please select all that apply = Treatment-oriented customisation (e.g. options to customise different exercises delivered through the app)

Or Did you use any customisation options provided through the app? Please select all that apply = Data visualisation customisation (e.g. option to compare data, view different visualisations of data entries etc.)

Q12 What customisation options did you value most and why? Please explain:

____

____

____

____

____

Display This Question:

If Which application/s did you download to use? Please select all that apply = Wysa

Or Which application/s did you download to use? Please select all that apply = Sanvello

Q13 Did you consider subscribing to the premium version of the app?

○ Yes

○ No

Display This Question:

If Did you consider subscribing to the premium version of the app? = Yes

Q14 What are the reasons for considering to upgrade your subscription?

____

____

____

____

____

Display This Question:

If Did you consider subscribing to the premium version of the app? = Yes

Q15 If you have already purchased an upgrade, tell us about your experience with the premium version of the app

____

____

____

____

____

Display This Question:

If Which application/s did you download to use? Please select all that apply = Sanvello

Q16 Did you use the discussion or chat groups today?

- Yes
- No

Display This Question:

If Did you use the discussion or chat groups today? = Yes

Q17 What are the reasons for joining the discussions and/or chat groups today?

____

Display This Question:

If Did you use the discussion or chat groups today? = Yes

Q18 How was your experience with the discussion and/or chat groups?

____

Display This Question:

If Which application/s did you download to use? Please select all that apply = Sanvello

Q19 Did you complete any modules today?

- Yes
- No

Display This Question:

If Did you complete any modules today? = Yes

Q20 A user upgrades level by completing modules. How do you find the idea of upgrading levels after completing modules?

____

Display This Question:

If Which application/s did you download to use? Please select all that apply = Wysa

Or Which application/s did you download to use? Please select all that apply = Sanvello

Q21 Did you share with anyone any information you recorded in the app?

- ○ Yes
- ○ No

Display This Question:

If Did you share with anyone any information you recorded in the app? = Yes

Q22 With whom do you/did you share the information you recorded through the app? (select all that apply)

- ▢ With my friends
- ▢ With family
- ▢ With my partner
- ▢ Other

Display This Question:

If With whom do you/did you share the information you recorded through the app? (select all that apply) = Other

Q23 Please specify your answer:

____

Q24 How do you remember to use the app? (select all that apply)

- ☐ I receive reminders from the app
- ☐ I have my own reminders (e.g. in a separate reminder app)
- ☐ I receive notifications when I don't use the app for a while
- ☐ It's part of my daily routine
- ☐ I do it automatically
- ☐ I do it in response to events or sensations (e.g. to stop a panic attack or reduce anxiety)
- ☐ I receive an email reminder from the research team
- ☐ Other

Q25 What did you like most about your interactions with the app/s today?

____

Q26 What did you dislike about your interactions with the app/s today?

____

Q27 If you could suggest three features to add or improve the application what would these be?

____

Online Questionnaire – Day 14

Q1 Which application/s did you download to use? Please select all that apply

- ☐ Sanvello
- ☐ Wysa
- ☐ Woebot

Q2 Did you use the application/s today?

- ○ Yes
- ○ No

Q3 Why did you not use the application/s today?

____

Q4 Please open the application/s now and use it for a while before filling the rest of the questionnaire

Q5 Roughly how much time did you spend using the application today?

- ○ 0-15 minutes
- ○ 15-30 minutes
- ○ 30-45 minutes
- ○ 45-60 minutes
- ○ More than an hour

Q6 What app features did you use today? Select all that apply:

- ☐ Mental Health Screening (questionnaires used to exam your emotional health)
- ☐ Self-monitoring (track mood and/or other factors related to your mood)
- ☐ Therapeutic elements (information about mental health, exercises designed to help alleviate anxiety symptoms)
- ☐ Immediate support (on-demand support provided when indicated you are in immediate need of help)
- ☐ Data visualisation (graphs or charts of your mood, comparison of mood related data)

Q7 What are the reasons for using these functions today?

____

____

____

____

____

Q8 Which options did you use to receive content through the app based on your inputs? Please select all that apply:

- ☐ I selected goal/s or challenge/s to work on when launching the app for first time
- ☐ I received recommendations of exercises to practice based on my mood entries
- ☐ I set up the notification timing
- ☐ None of the above

Q9 Were you satisfied with the content provided to you based on your inputs?

- ○ Yes
- ○ No

Q10 Please explain why the content provided to you based on your inputs was effective/ineffective:

____

____

____

____

____

Q11 Did you use any customisation options provided through the app? Please select all that apply

- ☐ Interface customisation (e.g. option to customize background theme, select nickname etc.)
- ☐ Treatment-oriented customisation (e.g. options to customise different exercises delivered through the app)
- ☐ Data visualisation customisation (e.g. option to compare data, view different visualisations of data entries etc.)
- ☐ I did not customise my app today

Q12 What customisation options did you value most and why? Please explain:

____

____

____

____

____

Q13 Did you consider subscribing to the premium version of the app?

- ○ Yes
- ○ No

Q14 What are the reasons for considering to upgrade your subscription?

____

____

____

Q15 If you have already purchased an upgrade, tell us about your experience with the premium version of the app

Q16 Did you use the discussion or chat groups today?

- ○ Yes
- ○ No

Q17 What are the reasons for joining the discussions and/or chat groups today?

Q18 How was your experience with the discussion and/or chat groups?

Q19 Did you complete any modules today?

- ○ Yes
- ○ No

Q20 A user upgrades level by completing modules. How do you find the idea of upgrading levels after completing modules?

____

Q21 Did you share with anyone any information you recorded in the app?

- ○ Yes
- ○ No

Q22 With whom do you/did you share the information you recorded through the app? (select all that apply)

- ☐ With my friends
- ☐ With family
- ☐ With my partner
- ☐ Other

Q23 Please specify your answer:

____

Q24 How do you remember to use the app? (select all that apply)

- ○ I receive reminders from the app
- ○ I have my own reminders (e.g. in a separate reminder app)
- ○ I receive notifications when I don't use the app for a while
- ○ It's part of my daily routine
- ○ I do it automatically
- ○ I do it in response to events or sensations (e.g. to stop a panic attack or reduce anxiety)
- ○ I receive an email reminder from the research team
- ○ Other

Q25 What did you like most about your interactions with the app/s today?

____

Q26 What did you dislike about your interactions with the app/s today?

____

Q27 Using a scale of 1-4, with 1 being the least and 4 being the most, please indicate which of the following features you used most the last 2 weeks? Select N/A if the app component is irrelevant:

	1 - the least used	2	3	4 - the most used	N/A
Mental Health Screening (questionnaires used to exam your emotional health)	○	○	○	○	○
Self-monitoring (track mood and/or factors related to your mood)	○	○	○	○	○
Therapeutic elements (features that help alleviate anxiety symptoms)	○	○	○	○	○
Immediate support (on-demand support provided when a need of immediate support is indicated)	○	○	○	○	○
Social features (peer or professional support offered through the app or through links to online services)	○	○	○	○	○
Data visualisation (visual presentation of past entries or other info entered through the app)	○	○	○	○	○

Q28 If you could improve the application, please give a brief description of how you would do so:

____

Start of Block: Section A - uMARS

Q29 Entertainment: Is the app fun/entertaining to use? Does it have components that make it more fun than other similar apps?

- ○ Dull, not fun or entertaining at all
- ○ Mostly boring
- ○ OK, fun enough to entertain user for a brief time (< 5 minutes)
- ○ Moderately fun and entertaining, would entertain user for some time (5-10 minutes total)
- ○ Highly entertaining and fun, would stimulate repeat use

Q30 Interest: Is the app interesting to use? Does it present its information in an interesting way compared to other similar apps?

- ○ Not interesting at all
- ○ Mostly uninteresting
- ○ OK, neither interesting nor uninteresting; would engage user for a brief time (< 5 minutes)
- ○ Moderately interesting; would engage user for some time (5-10 minutes total)
- ○ Very interesting, would engage user in repeat use

Q31 Customisation: Does it allow you to customise the settings and preferences that you would like to (e.g. sound, content and notifications)?

- ◯ Does not allow any customisation or requires setting to be input every time
- ◯ Allows little customisation and that limits app’s functions
- ◯ Basic customisation to function adequately
- ◯ Allows numerous options for customisation
- ◯ Allows complete tailoring the user’s characteristics/preferences, remembers all settings

---

Q32 Interactivity: Does it allow user input, provide feedback, contain prompts (reminders, sharing options, notifications, etc.)?

- ◯ No interactive features and/or no response to user input
- ◯ Some, but not enough interactive features which limits app’s functions
- ◯ Basic interactive features to function adequately
- ◯ Offers a variety of interactive features, feedback and user input options
- ◯ Very high level of responsiveness through interactive features, feedback and user input options

---

Q33 Target group: Is the app content (visuals, language, design) appropriate for the target audience?

- ◯ Completely inappropriate, unclear or confusing
- ◯ Mostly inappropriate, unclear or confusing
- ◯ Acceptable but not specifically designed for the target audience. May be inappropriate/unclear/confusing at times
- ◯ Designed for the target audience, with minor issues
- ◯ Designed specifically for the target audience, no issues found

Q34 Performance: How accurately/fast do the app features (functions) and components (buttons/menus) work?

- ◯ App is broken; no/insufficient/inaccurate response (e.g. crashes/bugs/broken features, etc.)
- ◯ Some functions work, but lagging or contains major technical problems
- ◯ App works overall. Some technical problems need fixing, or is slow at times
- ◯ Mostly functional with minor/negligible problems
- ◯ Perfect/timely response; no technical bugs found, or contains a ‘loading time left’ indicator (if relevant)

Q35 Ease of use: How easy is it to learn how to use the app; how clear are the menu labels, icons and instructions?

- ◯ No/limited instructions; menu labels, icons are confusing; complicated
- ◯ Takes a lot of time or effort
- ◯ Takes some time or effort
- ◯ Easy to learn (or has clear instructions)
- ◯ Able to use app immediately; intuitive; simple (no instructions needed)

Q36 Navigation: Does moving between screens make sense; Does app have all necessary links between screens?

- ◯ No logical connection between screens at all /navigation is difficult
- ◯ Understandable after a lot of time/effort
- ◯ Understandable after some time/effort
- ◯ Easy to understand/navigate
- ◯ Perfectly logical, easy, clear and intuitive screen flow throughout, and/or has shortcuts

---

Q37 Gestural design: Do taps/swipes/pinches/scrolls make sense? Are they consistent across all components/screens?

- ◯ Completely inconsistent/confusing
- ◯ Often inconsistent/confusing
- ◯ OK with some inconsistencies/confusing elements
- ◯ Mostly consistent/intuitive with negligible problems
- ◯ Perfectly consistent and intuitive

Q38 Layout: Is arrangement and size of buttons, icons, menus and content on the screen appropriate?

- ○ Very bad design, cluttered, some options impossible to select, locate, see or read
- ○ Bad design, random, unclear, some options difficult to select/locate/see/read
- ○ Satisfactory, few problems with selecting/locating/seeing/reading items
- ○ Mostly clear, able to select/locate/see/read items
- ○ Professional, simple, clear, orderly, logically organised

---

Q39 Graphics: How high is the quality/resolution of graphics used for buttons, icons, menus and content?

- ○ 1 Graphics appear amateur, very poor visual design - disproportionate, stylistically inconsistent
- ○ 2 Low quality/low resolution graphics; low quality visual design – disproportionate
- ○ 3 Moderate quality graphics and visual design (generally consistent in style)
- ○ 4 High quality/resolution graphics and visual design – mostly proportionate, consistent in style
- ○ 5 Very high quality/resolution graphics and visual design - proportionate, consistent in style throughout

---

Q40 Visual appeal: How good does the app look?

- ○ 1 Ugly, unpleasant to look at, poorly designed, clashing, mismatched colours
- ○ 2 Bad – poorly designed, bad use of colour, visually boring
- ○ 3 OK – average, neither pleasant, nor unpleasant
- ○ 4 Pleasant – seamless graphics – consistent and professionally designed
- ○ 5 Beautiful – very attractive, memorable, stands out; use of colour enhances app features/menus

Q41 Quality of information: Is app content correct, well written, and relevant to the goal/topic of the app?

- ○ N/A There is no information within the app
- ○ 1 Irrelevant/inappropriate/incoherent/incorrect
- ○ 2 Poor. Barely relevant/appropriate/coherent/may be incorrect
- ○ 3 Moderately relevant/appropriate/coherent/and appears correct
- ○ 4 Relevant/appropriate/coherent/correct
- ○ 5 Highly relevant, appropriate, coherent, and correct

Q42 Quantity of information: Is the information within the app comprehensive but concise?

- N/A There is no information within the app
- 1 Minimal or overwhelming
- 2 Insufficient or possibly overwhelming
- 3 OK but not comprehensive or concise
- 4 Offers a broad range of information, has some gaps or unnecessary detail; or has no links to
- more information and resources
- 5 Comprehensive and concise; contains links to more information and resources

Q43 Visual information: Is visual explanation of concepts – through charts/graphs/images/videos, etc. – clear, logical, correct?

- N/A There is no visual information within the app (e.g. it only contains audio, or text)
- 1 Completely unclear/confusing/wrong or necessary but missing
- 2 Mostly unclear/confusing/wrong
- 3 OK but often unclear/confusing/wrong
- 4 Mostly clear/logical/correct with negligible issues
- 5 Perfectly clear/logical/correct

Q44 Credibility of source: does the information within the app seem to come from a credible source?

- N/A There is no information within the app
- 1 Suspicious source
- 2 Lacks credibility
- 3 Not suspicious but legitimacy of source is unclear
- 4 Possibly comes from a legitimate source
- 5 Definitely comes from a legitimate/specialised source

Q45 Would you recommend this app to people who might benefit from it?

- 1 Not at all I would not recommend this app to anyone
- 2 There are very few people I would recommend this app to
- 3 Maybe There are several people I would recommend this app to
- 4 There are many people I would recommend this app to
- 5 Definitely I would recommend this app to everyone

Q46 How many times do you think you would use this app in the next 12 months if it was relevant to you?

- None
- 1-2
- 3-10
- 10-50
- >50

Q47 Would you pay for this app?

- 1 Definitely not
- 2
- 3
- 4
- 5 Definitely yes

Q48 What is your overall (star) rating of the app?

- 1 star - One of the worst apps I’ve used
- 2 stars
- 3 stars - Average
- 4 stars
- 5 stars - One of the best apps I've used

Q49 Using a scale of 1-5, with 1 being "Strongly disagree" and 5 being "Strongly agree", please indicate the impact of the app related to the targeted health behaviour (managing anxiety):

	Strongly disagree - 1	2	3	4	Strongly agree - 5
Awareness – This app has increased my awareness of the importance of addressing the health behaviour	○	○	○	○	○
Knowledge – This app has increased my knowledge/understanding of the health behaviour	○	○	○	○	○
Attitudes – The app has changed my attitudes toward improving this health behaviour	○	○	○	○	○
Intention to change – The app has increased my intentions/motivation to address this health behaviour	○	○	○	○	○
Help seeking – This app would encourage me to seek further help to address the health behaviour (if I needed it)	○	○	○	○	○
Behaviour change – Use of this app will increase/decrease the health behaviour	○	○	○	○	○

Q50 Further comments about the app?

____

____

____

____

Semi-structured Interviews Guide (sample questions)

Opening questions

1. Can you tell me a little about yourself? What do you do? What kind of technologies do you use in your daily life?
2. Can you tell me about the technologies you have used to support your mental health and well-being? Which apps or other sources of data? For how long? Roughly how much time did you spend using the application daily?
3. What were your expectations of using an app of this kind? How did you use the app?

Experience with app/s

4. Can you describe your overall experience with the app/s?

   - What aspects of the app were useful to you and why?
   - What features you liked and why?
   - What features you disliked and why?

5. Which are the positive aspects of your experience?

6. Which are the negative aspects of your experience?

Continued use/Abandonment

7. Why do you still use/did you stop using the application? Please explain.

Design of app/s – Suggestions for improvement

8. If you could suggest three features to add to or improve the application what would these be?

9. What could have made the app easier or more engaging? Please explain.

10. Overall based on your experience with the app, what are your suggestions for improvement, if any? Please explain.

11. Do you have any final thoughts you would like to share?
